# KIOM-79 Protects AGE-Induced Retinal Pericyte Apoptosis via Inhibition of NF-kappaB Activation In Vitro and In Vivo

**DOI:** 10.1371/journal.pone.0043591

**Published:** 2012-08-20

**Authors:** Junghyun Kim, Chan-Sik Kim, Eunjin Sohn, Yun Mi Lee, Kyuhyung Jo, Jin Sook Kim

**Affiliations:** Traditional Korean Medicine (TKM) Based Herbal Drug Research Group, Herbal Medicine Research Division, Korea Institute of Oriental Medicine, Daejeon, South Korea; University of Regensburg, Germany

## Abstract

KIOM-79 is an herbal mixture of parched Puerariae radix, gingered Magnoliae cortex, Glycyrrhizae radix and Euphorbiae radix. In the present study, we determined the efficacy and possible mechanism of KIOM-79 on the advanced glycation end product (AGE)-modified bovine serum albumin (BSA)-induced apoptosis of cultured bovine retinal pericytes and rat retinal pericytes in Zucker diabetic fatty (ZDF) rats. Seven-week-old male ZDF rats were treated with KIOM-79 (50 mg/kg body weight) once a day orally for 13 weeks. KIOM-79 significantly inhibited pericyte apoptosis which were induced by the AGE-BSA treatment. The KIOM-79 treatment markedly suppressed the activation of nuclear factor-kappaB (NF-κB) through the inhibition of inhibitory κB kinase complex. In addition, the oral administration of KIOM-79 inhibited the changes in retinal vasculature (vascular hyperpermeability, acellular capillary). KIOM-79 strongly inhibited pericyte apoptosis, NF-κB activation and the expression of pro-apoptotic Bax and tumor necrosis factor-α. Our results suggest that KIOM-79 may exert inhibitory effects on AGE-induced pericyte apoptosis by blocking NF-κB activation, thereby ameliorating retinal microvascular dysfunction.

## Introduction

Retinal microvascular cells undergo functional alterations and cell death under diabetic conditions [1]–[3]. The loss of retinal pericytes, a hallmark of early diabetic retinal changes, leads to the development of microaneurysms, retinal hemorrhages and neovasculization. The damage of retinal vessels causes permanent impairment of visual function. Advanced glycation end products (AGEs) are the late products of non-enzymatic glycation. The levels of these products are much higher in patients with diabetes [4]. Elevated AGEs levels closely correlate with the severity of diabetic retinopathy [5], [6]. In previous studies, it was shown that AGEs were accumulated in the retinal vascular cells of diabetic animals [7]. Administration of exogenous AGEs to non-diabetic animals induced thickening of the basement membrane of the retinal vessels [8], increased leukocyte adhesion [9] and increased breakdown of the blood retinal barrier [10]. Furthermore, it was reported that AGEs are also directly linked with the apoptotic cell death of retinal pericytes [1], [11], [12]. AGEs induced-apoptosis is mediated by increasing oxidative stress or via pro-apoptotic cytokine induced by interaction between AGEs and receptors for AGEs (RAGE) [13]–[15]. Recently, it was found that enhanced apoptosis of the retinal pericyte is also associated with nuclear factor (NF)-κB [16], [17]. NF-κB activation due to hyperglycemia induces accelerated pericyte loss [16].

KIOM-79 is a mixture of four herbal medicines, parched Puerariae radix, gingered Magnoliae cortex, Glycyrrhized radix and Euphorbiae radix, which are widely used for the treatment of diabetes or diabetic complications [18]–[20]. In previous studies, we reported that KIOM-79 inhibits the formation of AGEs in vitro, reduces the accumulation of AGEs in the kidneys of streptozotocin-induced diabetic rats [21] and prevents the development of diabetic nephropathy in non-obese type II diabetic Goto-Kakizaki rats [22]. KIOM-79 was also shown to inhibit the generation of reactive oxygen species (ROS) in a rat pancreatic beta-cell [23] and suppress the expression of vascular endothelial growth factor by high glucose in human retinal pigment epithelial cells [24].

Despite the various effects of KIOM-79 on diabetic complications, knowledge of its action mechanism and the effect on diabetic retinopathy is limited. To elucidate this issue, we investigated the anti-apoptotic and retinoprotective effects of KIOM-79 using bovine retinal pericytes and Zucker diabetic fatty (ZDF) rat, an animal model of type II diabetes. We determined the possible mechanism of KIOM-79 on NF-κB activation associated with the loss of retinal pericytes and identified the anti-apoptotic property of KIOM-79.

## Results

### HPLC Analysis of KIOM-79

**Figure 1:**
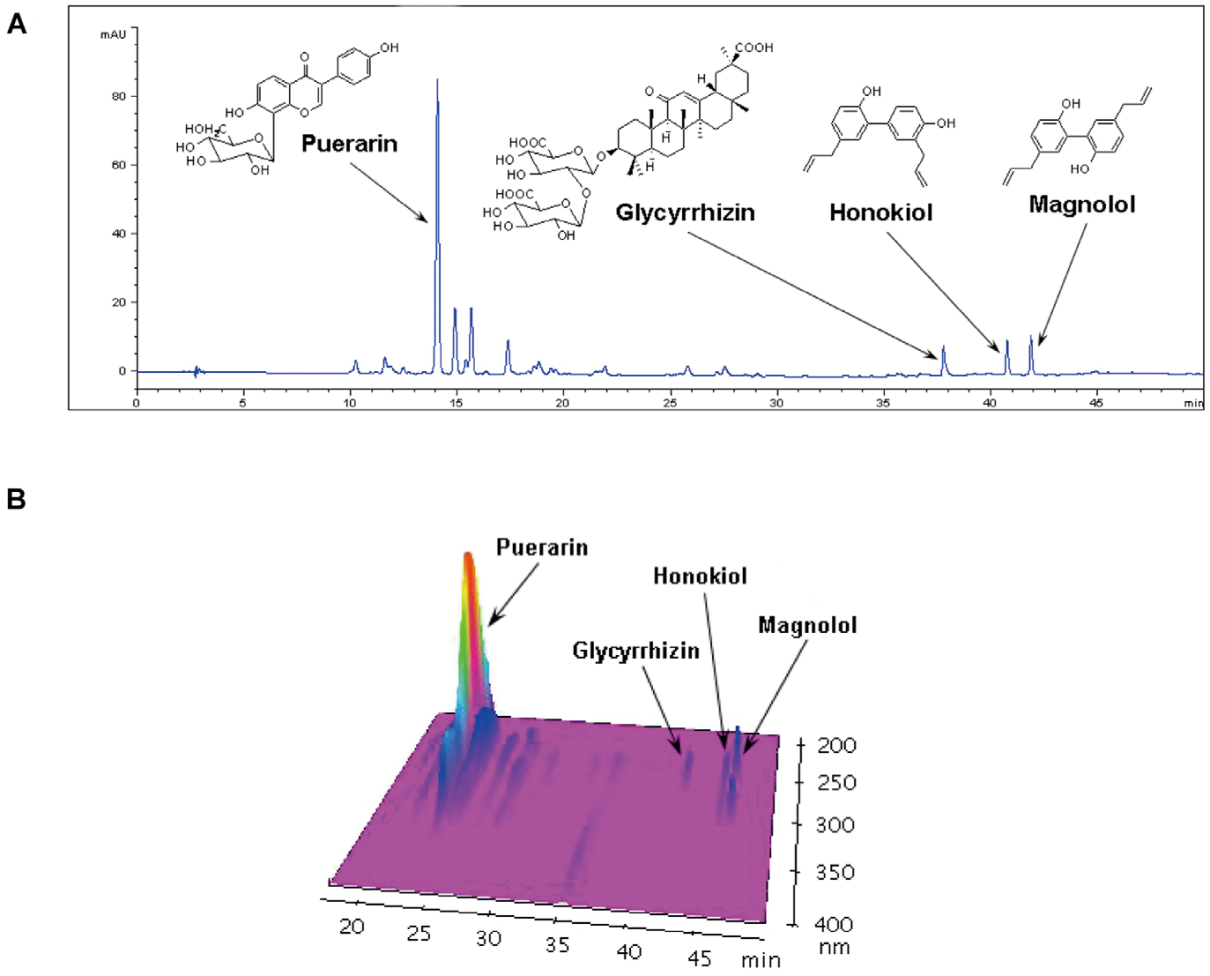
Analysis of KIOM-79 by HPLC. HPLC chromatogram of KIOM-79 by Agilent 1200 HPLC system with MWD detection at 254 nm (A) and 3D-HPLC chromatogram of KIOM-79 by Shimadzu HPLC system with DAD detection at 200–400 nm (B).

To certify the quality of KIOM-79, we performed HPLC analysis. Major compounds of KIOM-79 were puerarin, 3′-methoxypuerarin, puerarin-6″-*O*-*β*-apiofuranoside, daidzin, glycyrrhizine, honokiol and magnolol (Fig. 1).

### KIOM-79 Inhibits Pericyte Apoptosis Induced by AGE-BSA

**Figure 2:**
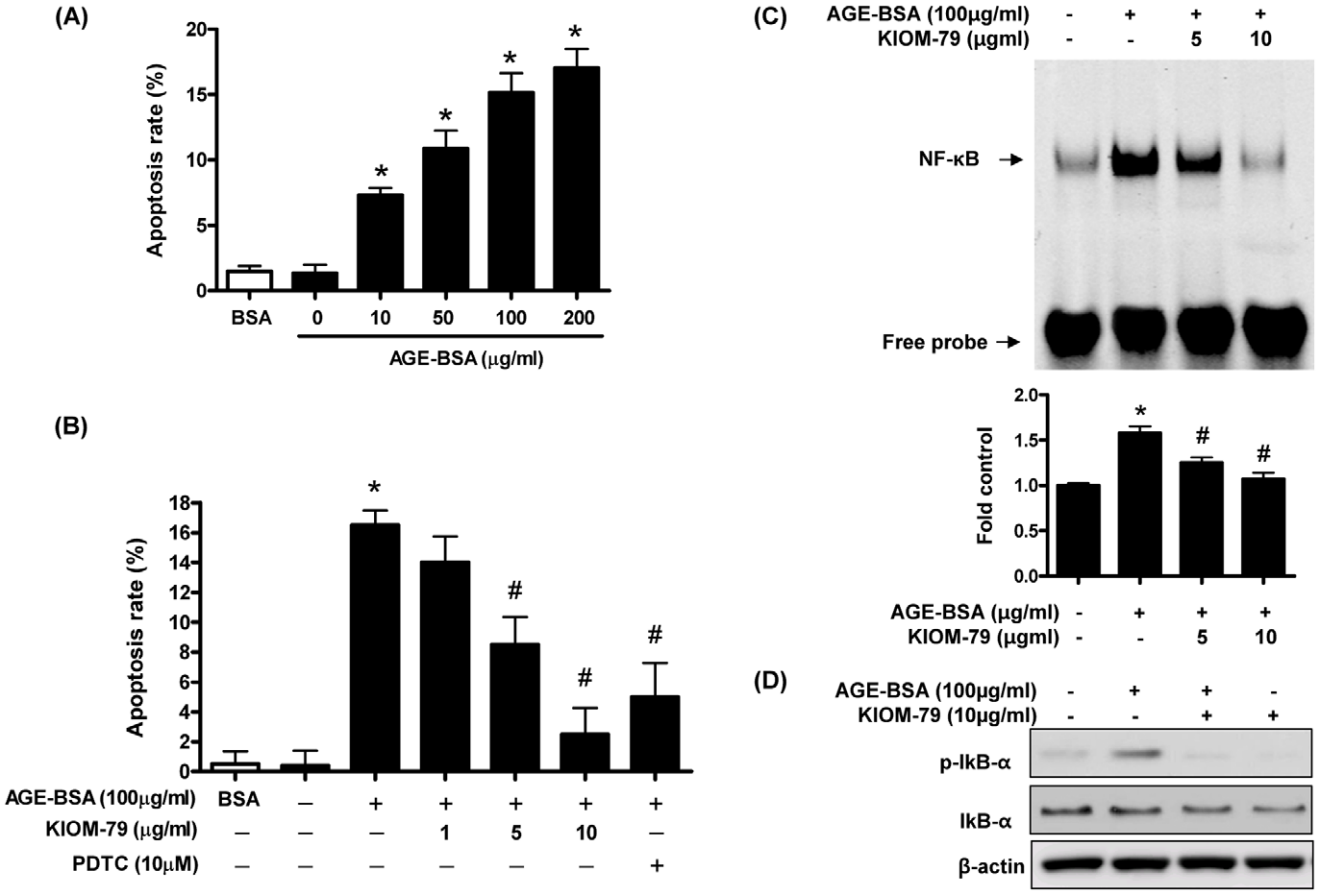
The effect of KIOM-79 on NF-κB activity in AGE-BSA-treated retinal pericytes. The pericytes were exposed for 6 hours to AGE-BSA (10 to 200 µg/mL) or BSA (A). The pericytes were pretreated with KIOM-79 or PDTC for 1 hour, followed by treatment with 100 µg/mL AGE-BSA for 6 hours (B–D). Apoptotic cells were detected using an FITC-labelled Annexin V protein and flow cytometry (A and B). Electrophoretic mobility shift assay for NF-κB (C). Western blot analysis was used to detect phospho-IκB-α and IκB-α (D). Each bar represents the mean ± SE from four independent experiments (*p<0.01 vs. control, ^#^p<0.01 vs. AGE-BSA).

The retinal pericyte apoptosis induced by AGE-modified bovine serum albumin (BSA) was examined in the presence of various concentrations of AGE-BSA (0, 10, 50, 100 and 200 µg/mL) for 6 hours (Fig. 2A). It was obvious that AGE-BSA induced pericyte apoptosis in a dose-dependent manner. The optimal response was achieved at 100 µg/mL AGE-BSA. A relatively small increase in apoptosis was observed when the concentration of AGE-BSA was doubled from 100 to 200 µg/mL. For all subsequent experiments, the pericytes were treated with 100 µg/mL AGE-BSA for 6 hours. To observe the effects of KIOM-79 on AGE-BSA-induced pericyte apoptosis, various concentrations of KIOM-79 (1, 5 and 10 µg/mL) were added 1 hour before the pericytes were stimulated for 6 hours with 100 µg/mL AGE-BSA. As shown in Fig. 2B and S1, KIOM-79 inhibited AGE-BSA-induced pericyte apoptosis in a dose-dependent manner, and the maximal inhibitory effect was observed at 10 µg/mL. To determine whether the inhibitory role of KIOM-79 in AGE-BSA-induced pericyte apoptosis involved NF-κB, we observed the effect of an NF-κB inhibitor (pyrrolidine dithiocarbamate, PDTC, 10 µM) on AGE-BSA-induced pericyte apoptosis (Fig. 2B and S1).

### Blockade of NF-κB Activation by KIOM-79

Since inhibition of NF-κB activity is considered one of the mechanisms promoting the apoptosis of retinal pericytes, we examined whether KIOM-79 inhibited the activation of NF-κB and the phosphorylation of IκB-α. EMSA analysis of the nuclear protein revealed a consistently increased level in the DNA binding activity of NF-κB after the 6-hour AGE-BSA treatment (a 1.8-fold increase versus the control; p<0.01; Fig. 2C). KIOM-79 significantly inhibited the DNA binding activity of NF-κB. As shown in Fig. 2D, KIOM-79 inhibited the phosphorylation of IκB-α in retinal pericytes.

### KIOM-79 Directly Inhibits IκB Kinase Complex (IKK) Activity

**Figure 3:**
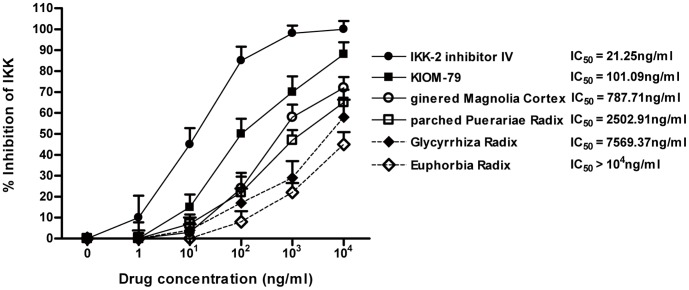
Effect of KIOM-79 on IκB kinase (IKK) complex activation. KIOM-79, parched Puerariae radix, gingered Magnoliae cortex, Glycyrrhized radix and Euphorbiae radix, and the IKK-2 inhibitor IV supplied by the manufacturer in this kit were tested for their ability to inhibit IKK-b activity using an ELISA-based kinase activity assay. Inhibition by a compound was defined by the 50% inhibition concentration (IC_50_) of the IKK activity. IC_50_ values were calculated from the dose inhibition curve. Values in the graphs represent means ± SE, n = 6.

NF-κB activation requires phosphorylation of the inhibitory protein IκB-α by IκB kinase [25]. On the basis of the above-mentioned results, it was assumed that the NF-κB inhibitory effects of KIOM-79 are due to modulation of IKK complex. In order to establish whether KIOM-79 affects IKK activity, an ELISA-based IKK activity assay was applied. KIOM-79 and each herbal extract dose-dependently inhibited the activity of IKK complex. Furthermore, KIOM-79 exhibited a stronger effect, compared to its single component herb (Fig. 3). These results suggest that KIOM-79 might be a kinase inhibitor and that the four herbs act synergistically.

### Prevention of Histopathological Changes in the Retina by KIOM-79

**Figure 4:**
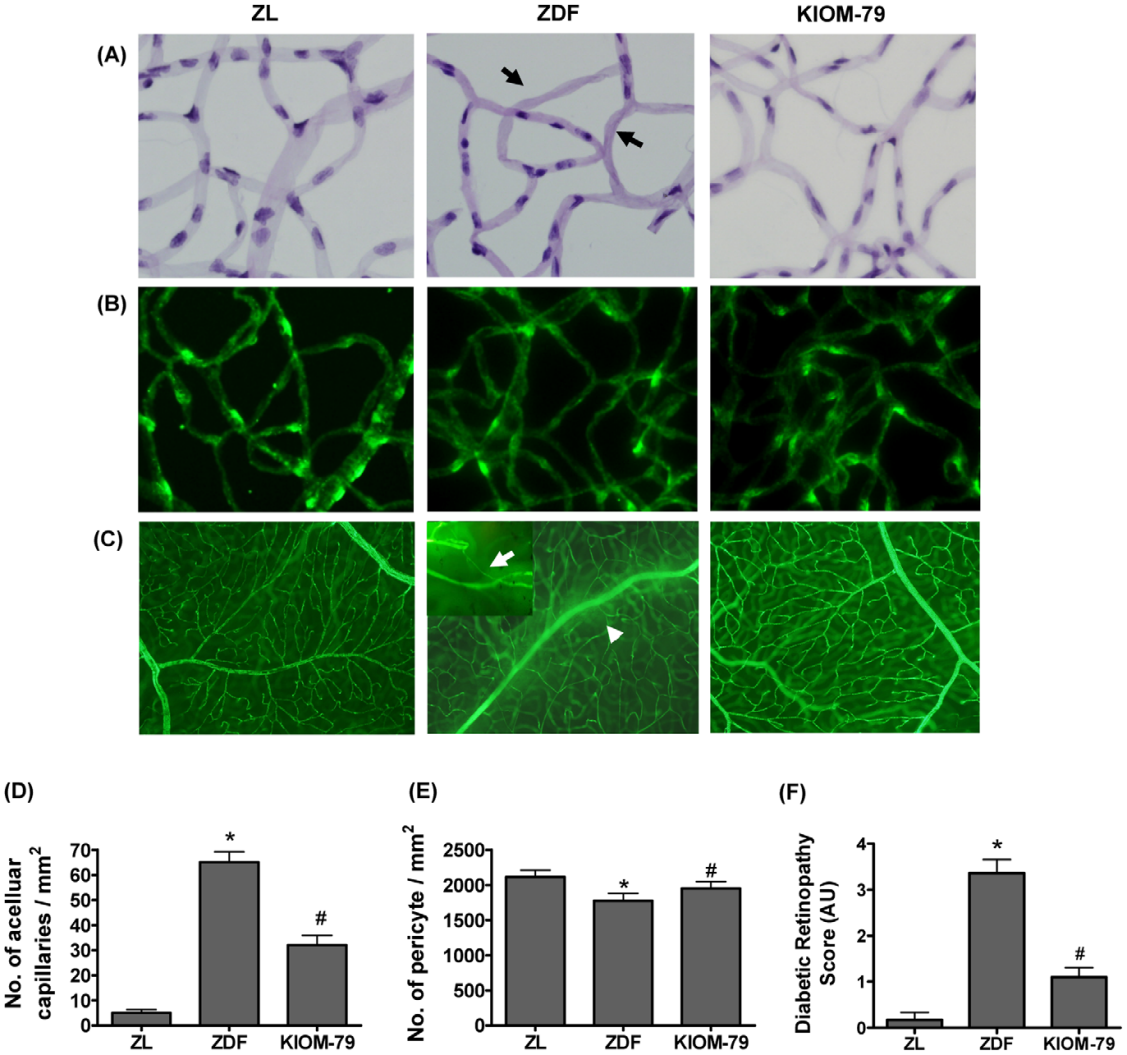
Effect of KIOM-79 on histopathological changes. (A) Trypsin-digested retinal vessel pattern analysis. Representative whole mount of retinal vessel from a normal Zucker lean rat (ZL), vehicle-treated ZDF rat (ZDF) and ZDF rat treated with KIOM-79 (KIOM-79) was stained with periodic acid-Schiff. Acellular capillary (black arrow) were observed in vehicle-treated ZDF rats. X200 magnification. (B) Immunofluorescence staining for NG2 (green) in trypsin-digested retinal vessels. (C) In fluorescein-dextran microscopy, the white arrowhead and arrow indicate the fluorescein leakage areas and vessel narrowing (magnified inset), respectively. For quantitative analysis, (D) acellular formation of retinal vessel were counted, and (E) the number of pericytes was determined by counting the number of NG2 positive cells per mm^2^ of capillary area. (F) The changes in retinal angiography lesion scores obtained using the retinal scoring methods described in Materials and Methods. Values in the bar graphs represent means ± SE, n = 8. *p<0.01 vs. normal ZL rats, ^#^p<0.01 vs. vehicle-treated ZDF rats.

At 21 weeks of age, all ZDF rats were developed hyperglycemia compared to the normal ZL rat. As shown in Table 1, the untreated ZDF rats had a more than four-fold increase of fasting blood glucose level. Body weights of the untreated ZDF rats were elevated to approximately 78% compared to the normal ZL rats. KIOM-79 induced a minor decrease of blood glucose levels, but not affected on HbA1c level and body weight. Diabetic retinopathy induces several histopathological changes, such as blood-retinal barrier breakdown, acellular capillary formation and ganglion cell loss [3], [26]. Therefore, we evaluated the effects of KIOM-79 on these histopathological changes using the following three histological evaluations. First, we used retinal digest preparations to determine the presence of acellular capillary. The vehicle-treated ZDF rats showed a small increase in the numbers of acellular capillaries compared to the normal ZL rats. However, treatment with KIOM-79 significantly inhibited these changes (Fig. 4A and 4D). Immunohistofluorescence analysis for NG2, which is a marker for pericytes, showed diabetes-induced pericyte loss in the vehicle-treated ZDF rats. In the normal ZL rats, the NG2 immunoreactivity was concentrated in the cell body of the pericytes (Fig. 4B). Analysis of the number of NG2-positive cell bodies on the capillaries revealed that the pericyte density significantly decreased in the vehicle-treated ZDF rats. However, the treatment with KIOM-79 significantly inhibited the pericyte loss (Fig. 4E, p<0.01). Next, we evaluated retinal vessel dysfunction using fluorescein-dextran microscopy. After fluorescein-dextran perfusion, the retinal vessels from normal ZL rats could be clearly delineated with no leakage into the neural retina. By contrast, vehicle-treated ZDF rats demonstrated a marked retinal vascular leakage as evidenced by widespread, diffuse fluorescence and reduced occludin expression in the retinal capillaries, and the numbers of abnormal vessels showing vessel narrowing and capillary non-perfusion were increased (Fig. 4C and S2). However, KIOM-79-treated ZDF rats had significantly lower scores than that of vehicle-treated ZDF rats by quantitative lesion scoring, (Fig. 4F).

**Table 1:** Metabolic and physical parameters.

	ZL	ZDF	KIOM-79
Body weight (g)	349.9±15.1	447.9±24.7*	437.6±20.3
Blood glucose (mg/dl)	92.9±4.1	489.8±17.0*	391.7±42.8†
HbA1c (%)	3.7±0.1	7.88±0.4*	7.1±0.3

ZL: normal Zucker lean rats, ZDF: vehicle-treated Zucker diabetic fatty rats, KIOM-79: Zucker diabetic fatty rats treated with KIOM-79 (50 mg/kg body weight). All data were expressed as mean ± SE.

* p<0.01 vs. normal ZL rats,

† p<0.05 vs. untreated ZDF rats.

### KIOM-79 Inhibits AGEs Formation

**Figure 5:**
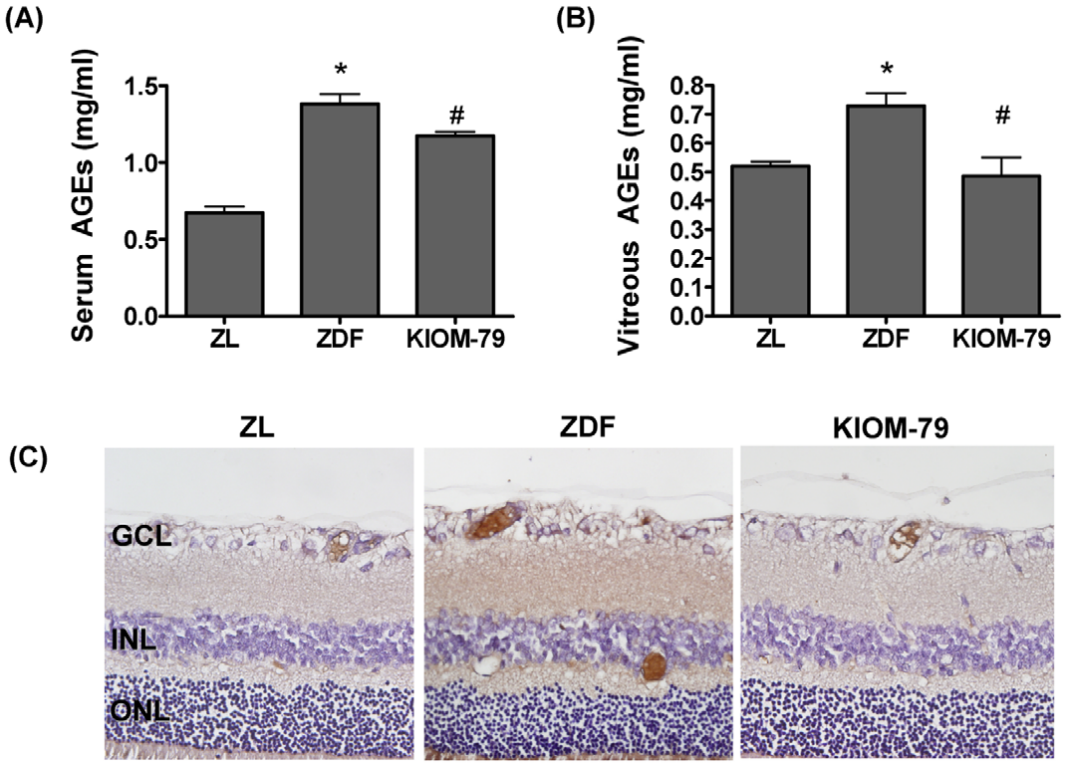
Effect of KIOM-79 on the formation of advanced glycation end products (AGEs). AGEs levels in (A) blood and (B) vitreous were obtained using ELISA-based assays. The values in the graph represent means ± SE, n = 8. *p<0.01 vs. normal ZL rats, ^#^p<0.01 vs. vehicle-treated ZDF rats. (C) Representative immunostaining of AGEs in retinas. The sections were visualized by 3,3′-diaminobenzidine tetrahydrochloride substrate staining (brown) and counterstained with Mayer’s hematoxylin.

KIOM-79 was tested for its ability to inhibit AGEs formation and accumulation in the retina. At the end of the study, the AGEs levels in both serum and vitreous were remarkably elevated in vehicle-treated ZDF rats compared to normal ZL rats. However, these levels in the KIOM-79-treated ZDF rats were significantly decreased compared to vehicle-treated ZDF rats (Fig. 5A and B). We next carried out immunohistochemical staining for AGEs. It was apparent that AGEs immunoreactivity was only contained in the large and small retinal vessels of the normal ZL rats, whereas AGEs-positive signals were located in both the retinal vessels and the inner neural retina in the vehicle-treated ZDF rats, indicating that serum AGEs had accumulated in the retinal tissues. However, treatment with KIOM-79 reduced the AGEs deposited in these regions (Fig. 5C).

### KIOM-79 Prevents the Apoptosis of Retinal Pericytes in ZDF Rats

**Figure 6:**
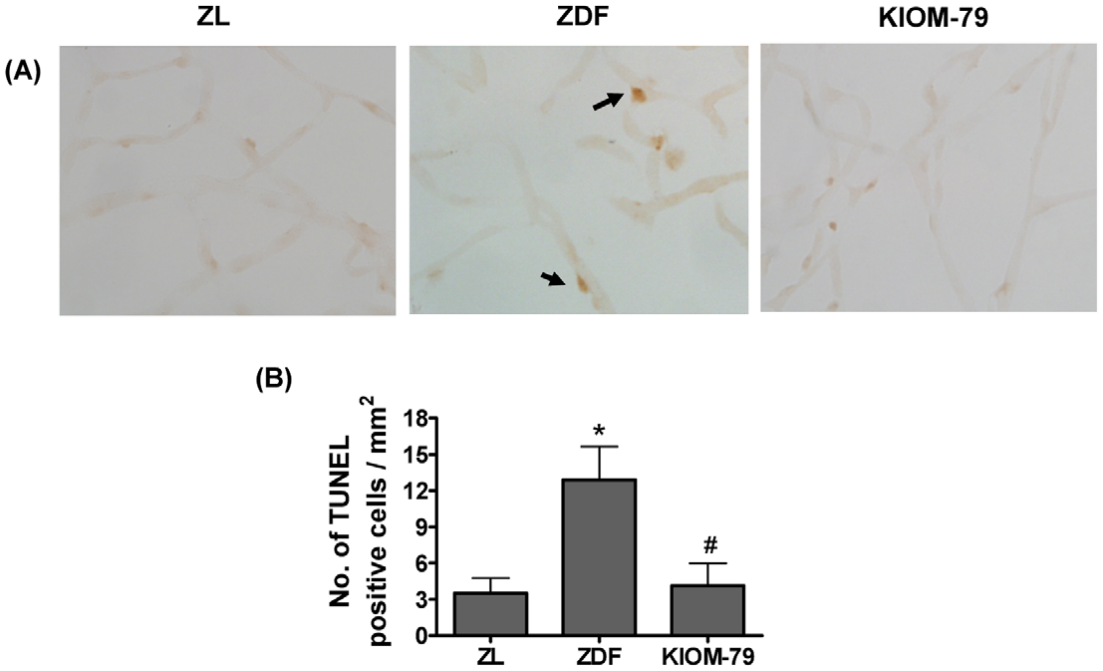
Apoptosis of retinal pericytes. (A) Representative trypsin-digested retinal vessels from a normal Zucker lean rat (ZL), vehicle-treated ZDF rat (ZDF) and ZDF rat treated with KIOM-79 (KIOM-79) were stained with TUNEL (brown). Apoptotic pericytes (arrow) were observed in vehicle-treated ZDF rats. X200 magnification. (B) Quantitative analysis of TUNEL-positive cells in trypsin-digested retinal vessel. All data are expressed as mean ± SE, n = 8. *p<0.01 vs. normal ZL rats, ^#^p<0.01 vs. vehicle-treated ZDF rats.

To characterize the death of pericytes in the vehicle-treated ZDF rats, we applied the apoptosis assay. Examination of the retinal trypsin digests of vehicle-treated ZDF rats showed many TUNEL-positive pericytes and endothelial cells in the retinal vessels, whereas normal ZL rats and KIOM-79 treated ZDF rats had few positive cells (Fig. 6). These results suggest that several pericytes were undergoing apoptosis under diabetic conditions, which might lead to histopathological changes, such as the acellular formation and the vascular leakage. KIOM-79 had an anti-apoptotic effect on these cells.

### KIOM-79 Inhibits the Activation of NF-κB in the Retina

**Figure 7:**
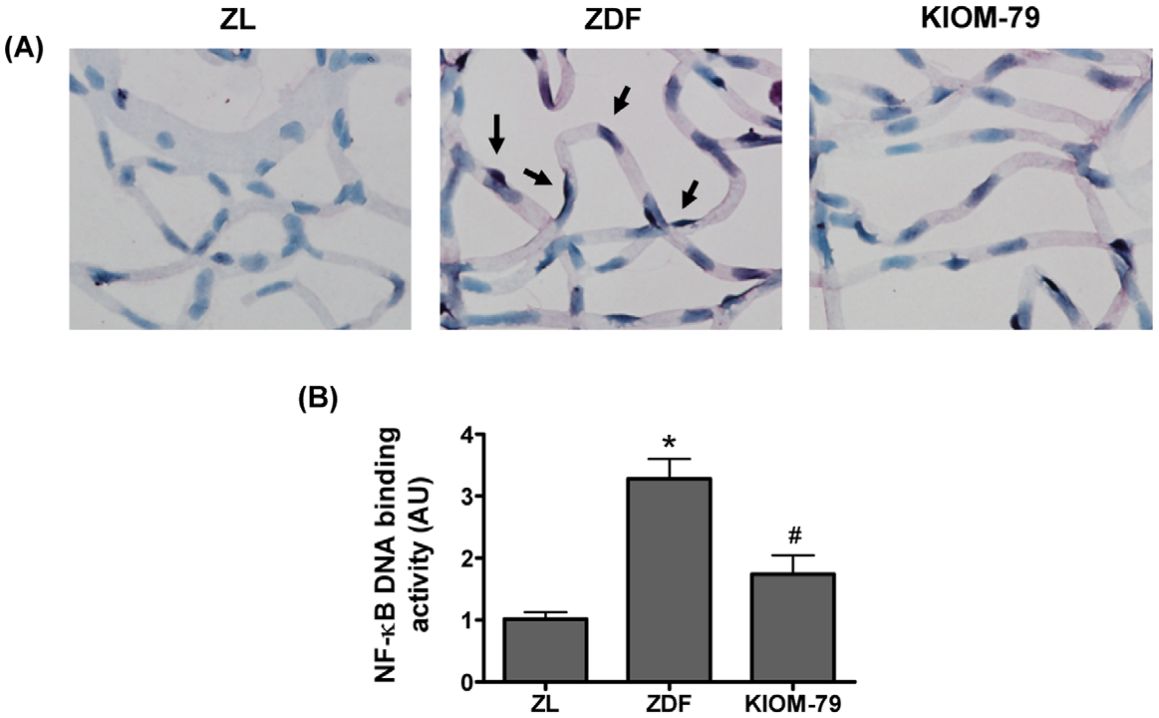
Effect of KIOM-79 on NF-κB activation. (A) Representative photomicrographs of retinal vessels from a normal Zucker lean rat (ZL), vehicle-treated ZDF rat (ZDF) and ZDF rat treated with KIOM-79 (KIOM-79). NF-κB activation (arrow) was determined by southwestern histochemistry. The sections were visualized by nitroblue tetrazolium chloride (NBT) and 5-bromo-4-chloro-3-indolyl phosphate (BCIP) substrate staining and counterstained with methyl green X200 magnification. (B) Analysis of NF-κB DNA binding activity by ELISA-based assay. KIOM-79 significantly inhibited nuclear NF-κB DNA binding activity (p<0.01). Values in the bar graphs represent means ± SE, n = 8. *p<0.01 vs. normal ZL rats, ^#^p<0.01 vs. vehicle-treated ZDF rats.

NF-κB activity was detected in retinal vessels by southwestern histochemistry. This technique allows the localization of activated nuclear factor in the cellular nucleus. Using this novel method, we observed that marked NF-κB activity was mainly found in the nuclei in retinal pericytes and endothelial cells in the vehicle-treated ZDF rats. KIOM-79 inhibited the expression of activated NF-κB (Fig. 7A). To confirm the results of southwestern histochemistry and evaluate NF-κB activation in a quantitative way, we also performed an ELISA-based NF-κB assay. Similarly, vehicle-treated ZDF rats presented a significantly higher activity of NF-κB than normal ZL rats, whereas the levels of activated NF-κB in KIOM-79-treated ZDF rats were significantly lower (by 50%) than those of vehicle-treated ZDF rats (Fig. 7B).

### KIOM-79 Inhibits the Expression of Pro-apoptotic Molecules

**Figure 8:**
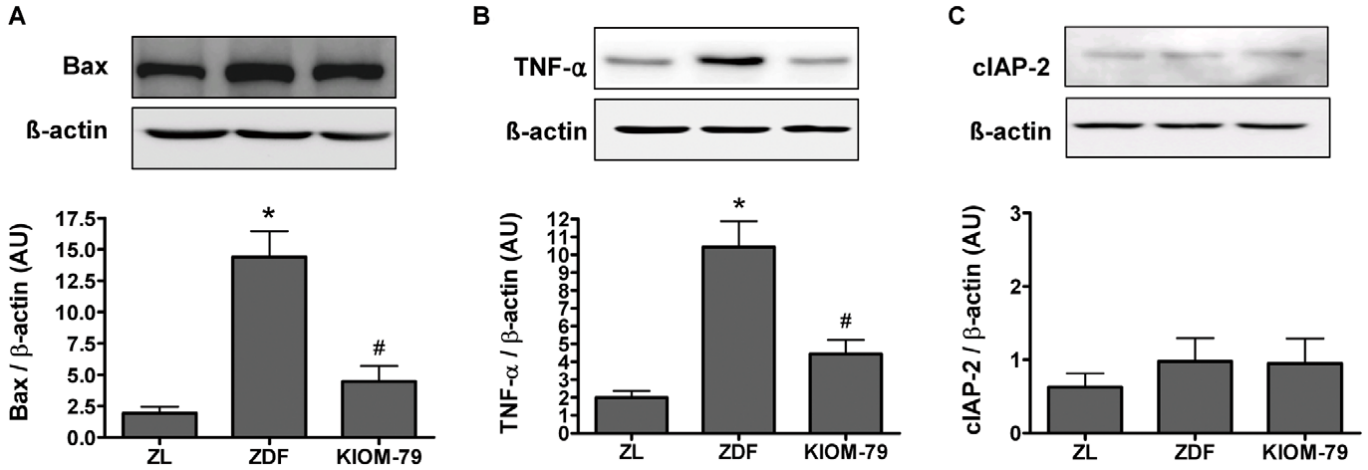
Protein expression of Bax (A), TNF-α (B) and cIAP-2 (C). Western blot analysis in retina tissue from a normal Zucker lean rat (ZL), vehicle-treated ZDF rat (ZDF) and ZDF rat treated with KIOM-79 (KIOM-79). Values in the bar graphs represent means ± SE, n = 8. *p<0.01 vs. normal ZL rats, ^#^p<0.01 vs. vehicle-treated ZDF rats.

To further investigate the pro-apoptotic role of NF-κB and the anti-apoptotic effect of KIOM-79, we focused on the expression of NF-κB-induced pro-apoptotic molecules, such as Bax [27] and TNF-α [28] and NF-κB-induced anti-apoptotic molecules, such as cIAP-2 [29]. Western blot analysis revealed that the enhanced expression of Bax and TNF-α were detected in the vehicle-treated ZDF rats, while it was significantly inhibited in KIOM-79-treated ZDF rats (Fig. 8A and 8B). On the other hand, the expression level of cIAP-2 was not changed in vehicle-treated ZDF rats (Fig. 8C). It thus seemed that activated NF-κB in the retina under diabetic conditions has a pro-apoptotic role and KIOM-79 has an anti-apoptotic effect via inhibition of the activation of NF-κB.

## Discussion

Medicinal herbs are rich sources of potential preventive and therapeutic agents against diabetes or its complications, but require detailed and systematic in vitro and in vivo evaluations. In the present study, we examined the anti-apoptotic effect of an herbal medicine, KIOM-79.

One of the major consequences of hyperglycemia is the formation of AGEs. AGEs have been implicated in the pathogenesis of diabetic retinopathy, and inhibiting the formation of AGEs can improve diabetic retinopathy [12], [30], [31]. In addition, the interaction between AGE and RAGE elicits the generation of reactive oxygen species and the expression of proinflammatory cytokines [12], [30]. AGEs have also been directly linked with the apoptotic cell death of retinal pericytes [1], [11], [12]. AGE-induced apoptosis is mediated by increasing oxidative stress or via pro-apoptotic cytokines induced by the AGE/RAGE interaction [13], [14], [32]. Thus, the inhibition of AGEs formation or the blockade of the AGE-RAGE axis has been suggested as a novel therapeutic target for diabetic retinopathy [31]. Indeed, KIOM-79 had the property of AGEs inhibitor in retinal tissue. This result suggests that KIOM-79 treatment results in the decrease of interaction between AGEs and RAGE and a decline in cellular damage mediated by AGEs.

NF-κB is an important transcription factor and has been implicated in the development of diabetic retinopathy [16]. The hyperglycemic activation of NF-κB induces accelerated pericyte loss via the induction of NF-κB-controlled pro-apoptotic molecules [16]. We have demonstrated that AGEs stimulates the activation of NF-κB and that KIOM-79 can almost completely inhibit this activation. KIOM-79 may have the properties of NF-κB inhibitor. KIOM-79 was originally reported as an inhibitor of AGEs formation *in vitro* [21], [22]. However, Kang et al. observed that KIOM-79 also has cytoprotective effect in pancreatic beta-cells [23]. In present study, we demonstrated the anti-apoptotic effect of KIOM-79 in retinal pericytes. In the adult retina, the death of pericytes leads to the development of diabetic retinopathy because pericytes can not replicate [26]. Interestingly, it was reported that the accumulation of AGEs in pericytes induced apoptosis [1], [11], [12], [33]. Moreover, AGEs interact with RAGE, inducing subsequent activation of NF-κB and NF-κB-controlled pro-apoptotic molecules. Our results suggest that anti-apoptotic effect of KIOM-79 is probably due to its inhibitory effect on NF-κB activation. Notably, the inhibitory effect of KIOM-79 on NF-κB activation might be due to their ability to inhibit IKK complex.

The destruction of retinal pericytes results in the formation of acellular capillaries, which are associated with areas of non-perfusion. The increasing of acellular capillaries then leads to the development of retinal ischemia and retinal neovascularization. Therefore, inhibition of the development of acellular capillaries by KIOM-79 could be expected to inhibit the development of retinal ischemia and neovascularization. Furthermore, the fluorescein angiography showed that KIOM-79 markedly inhibited the fluorescein leakage, which suggests that KIOM-79 might prevent the breakdown of blood-retinal barrier (BRB). Diabetes-induced occludin degradation was attenuated by the treatment of KIOM-79. The expression of occludin correlates with an increased function in the barrier. Although occludin by itself cannot form a functionally tight barrier, it likely plays an important role in the organization and stabilization of the tight junction. The results suggest that KIOM-79 has potential to prevent BRB dysfunction induced by diabetes.

Intensive glycemic control generally inhibited the progression of diabetic retinopathy [34]. However, it was difficult for many patients to maintain the optimal metabolic level. Thus, the development of new drug to modulate the mechanism involved in diabetic retinopathy is needed, even if the blood glucose was not controlled perfectly. KIOM-79 also had significant effects on any parameters of retinal structure and function without the strong reduction of blood glucose. These findings suggest that even in hyperglycemia, it is possible to attenuate retinal injury by KIOM-79. In addition, KIOM-79 demonstrated an augmented synergistic inhibitory effect on the activation of IKK complex compared to its individual herbal constituents. The synergistic effect of KIOM-79 was also shown in the production of nitrite in lipopolysaccharide-stimulated murine macrophages [35] and AGEs formation in vitro [21]. However, the reasons for this phenomenon are unclear. In many cases, single herbs are seldom used alone; herbal mixtures were known to have various advantages of synergy and interactions among the various phytocompounds present in the different herbs [36]. KIOM-79 was created based on the known functions of each four herbs used in traditional Korean medicine. In present study, we identified four major compounds (magnolol, honokiol, glycyrrhizine and puerarin) in KIOM-79. Each component is considered to have the anti-diabetic effects [37]–[40]. Therefore, the multifunctional activities of KIOM-79 against diabetic complications may be considered to be due to synergistic effects among the components in the four herbs.

In summary, KIOM-79 attenuates the AGEs-induced apoptosis of retinal pericytes through blocking NF-κB activation. KIOM-79 acts as an IKK inhibitor. Taken together, these results indicate that treatment with KIOM-79 could be a valuable therapeutic approach in diabetic retinopathy.

## Materials and Methods

### Ethics Statement

All animal procedures were performed in accordance with the ARVO Statement for the Use of Animals in Ophthalmic and Vision Research and approved by the Korea Institute of Oriental Medicine Institutional Animal Care and Use Committee. No specific permits were required for the described field studies. No specific permissions were required for these locations/activities. We confirmed that the location was not privately-owned or protected in any way and the field studies did not involve endangered or protected species.

### Preparation of KIOM-79

KIOM-79 was prepared as previously reported [22]–[24], [35]. Briefly, the cortex of *Magnolia officinalis*, radix of *Pueraria lobata*, radix of *Glycyrrhiza uralensis* and radix of *Euphorbia pekinensis* were collected from plants obtained from Gamsuk Province (China). Magnoliae cortex (100 g) was simmered with 3 g of Zingiberis rhizoma for 60 min. Puerariae radix (100 g) was stir-roasted at 75°C for 45 min and, when the radix surface yellowed with brown spots, it was removed and allowed to cool. Equal amounts of gingered Magnoliae cortex, parched Puerariae radix, Glycyrrhizae radix and Euphoriae radix were mixed, pulverized, extracted in 80% ethanol for one week at room temperature, concentrated with a rotary evaporator and lyophilized, and the entire procedure was repeated four times.

### HPLC Analysis of KIOM-79

To quantify major compounds of KIOM-79, the high-performance liquid chromatography (HPLC) analysis was performed by an Agilent 1200 HPLC system and the 3D-HPLC chromatogram was acquired by a Shimadzu HPLC system. Spherex C-18 analytical column (250×4.6 mm, 5.0 *µ*m, Phenomenex) was used with the mobile phase consisted of acetonitrile (*A*) and 0.1% acetic acid in water (*B*). The mobile phase gradient elution was programmed as follow: 95–70% *B* (0–30 min), 70–40% *B* (30–40 min), 40–0% *B* (40–45 min), 100% *A* (45–50 min), then a column washed with 100% *A* and returned to 95% *B*. The column temperature was maintained at 30, flow rate set at 1 ml/min, and sample injection volume was set at 10 µl. The multiple wavelength detector (G1365B) set at 254 nm and diode array detector (SPD-M10A) set at 200–400 nm.

### Primary Bovine Retinal Pericytes Culture

Primary retinal pericyte cells were isolated from bovine retinal microvessels using a modification of previous published methods [41]–[44]. Briefly, bovine eyes were obtained from a local slaughterhouse (Jangwon Food, Daejeon, South Korea) and transported on ice to the laboratory. Under aseptic conditions, the retinas were removed from the eyes and transferred to a 100 mm diameter petri dish filled with sterile phosphate-buffered saline. The pigment epithelium and choroid were carefully removed Retinas were rinsed thoroughly and then homogenized using a Telflon-glass homogenizer (Wheaton, Millville, NJ, USA) and filtered though a 70 micron nylon mesh. The remaining retentate was digested in 0.066% Collagenase/Dipase (Roche Applied Science, Mannheim, Germany) and 0.033% bovine serum albumin in DPBS for 1 hour at 37°C. The cellular digests were filtered through a 40 micron nylon mesh and then centrifuged. Purification of bovine retinal pericytes was achieved with CELLection Pan Mouse IgG Kit (Invitrogen Dynal AS, Oslo, Norway). The pellets were suspended in Dulbecco’s Modified Eagle’s Media (DMEM) containing 10% fetal bovine serum (FBS) and incubated with mouse anti-desmin monoclonal antibody (Millipore, Billerica, MA, USA) for 10 minutes at 4°C. After washing, the cells were incubated with Dynabeads for 20 minutes at 4°C with gentle rotation. The bead-bound pericytes were released and then resuspended in DMEM containing 20% FBS at 37°C in a humidified atmosphere of 5% CO_2_ incubator. After cell attachment, the medium was changed every 3 to 4 days with DMEM containing 10% FBS. Pericytes were identified by the inability to uptake rhodamine-conjugated, acetylated low-density lipoprotein as described by Cacicedo *et al*. [45]. Our cultured cells did not contain any cells that reacted with antibodies to the endothelial cell marker von Willebrand’s factor (Dako, CA, USA). Cells between passages 3 and 5 were used in this experiment. Cells were plated onto appropriate culture dishes and used for experiments upon reaching 80% confluence. Standard culture medium was replaced with fresh serum-free medium 16 h before experiments. Reagents used for cell culture were from GIBCO BRL (Grand island, NY, USA).

### Cellular Apoptosis Detected Using Flow Cytometry

Bovine retinal pericytes were seeded in 12-well plates. The cells were pre-incubated with KIOM-79 (1, 5 and 10 µg/ml) or PDTC (10 µM, Calbiochem, San Diego, CA, USA) for 1 h, and the retinal pericytes were stimulated with various concentrations of AGE-BSA (CircuLex, Nagano, Japan) for 6 h. Nonglycated BSA was used as a control to ensure that each control well was exposed to an albumin concentration equivalent to that of the treated wells. The cells were then collected and treated according to the protocol included in Annexin V-fluorescein isothiocyanate (FITC) Apoptosis Detection Kit (Merck, Darmstadt, Germany) and the percentages of apoptotic cells were determined using a flow cytometer (FACS Calibur, Becton Dickinson, San Jose, CA, USA). The results were analyzed using Cell Quest Pro software (Becton Dickinson).

### Measuring of NF-κB Activity

For an electrophoretic mobility shift assay (EMSA), nuclear extracts were prepared using a NE-PER kit (Pierce Biotechnology, Rockfold, IL, USA) according to the manufacturer’s instructions. The EMSA assay was performed by incubating 10 µg of nuclear protein extract with IRDye 700-labeled NF-κB oligonucleotide (LI-COR, Lincoln, NE, USA) or an unlabelled probe, which was used as a cold competitor. The EMSA gels were analyzed, and the images were captured and quantified using a LI-COR Odyssey infrared laser imaging system (LI-COR).

### Western Blotting

Western blotting was performed as previously described [46]. Primary antibodies against phospho-IkB-α (#9246), IkB-α (# 9242) were obtained from Cell Signaling Technology (Danvers, MA, USA). β-actin (A-1978) was obtained from Sigma (St. Louis, MO, USA).

### IκB Kinase Complex Assay

To determine whether KIOM-79 and its single component herb (ethanol extracts of parched Puerariae radix, gingered Magnoliae cortex, Glycyrrhized radix and Euphorbiae radix) affects IKK complex, IKK-β kinase activity was evaluated with an IKK-β-inhibitor screening kit (Calbiochem, CA, USA) according to the manufacturer’s instructions. The data were used to calculate the percentage inhibition and 50% inhibitory concentration (IC_50_) from a standard curve.

### Animals and Experimental Design

Male 6-week-old ZDF rats (ZDF/Gmi-fa/fa) and Zucker lean (ZL) counterparts (ZDF/Gmi-lean) were purchased from Charles River Laboratory (Waltham, MA, USA) and acclimated for 1 week prior to the study. Rats were individually housed in plastic cages and maintained at 24°C±2°C with a 12 h light:dark cycle and received a diet of Purina 5008 (Ralston Purina, St. Louis, MO, USA) and tap water *ad libitum*. Rats were divided into 3 groups of 8 rats according to their initial blood glucose concentration as follows: (1) normal ZL rats, (2) vehicle-treated ZDF rats and (3) ZDF rats treated with KIOM-79 (50 mg/kg body weight). KIOM-79 was dissolved in distilled water and administered once a day through oral gavage for 13 weeks. The blood glucose level and body weight were monitored consecutively, and glycated hemoglobin was determined by a commercial kit (Unimate HbA1c, Roche Diagnostics, Mannheim, Germany). At necropsy, the right eye from each rat was enucleated under deep anesthesia, following an intraperitoneal injection of pentobarbital sodium (30 mg/kg body weight) and fixed in 10% neutralized formalin for 24 h and embedded in paraffin. After the right eye was removed, 1 ml of phosphate buffered saline (PBS) containing 50 mg of fluorescein-dextran (Sigma, St. Louis, MO, USA) was injected into the left ventricle. After 10 min, the left eye of each rat was enucleated and cut into three sectors centered at the optic disc so that three retinal samples of similar size were obtained from each eye. The three retinal samples were subjected to trypsin-digested vessel preparation, fluorescein-dextran microscopy and extraction of protein, respectively.

### Trypsin-digested Vessel Preparation

The retinal samples were placed in 10% formalin for 2 days. After fixation, the retina was incubated in trypsin (3% in sodium phosphate buffer containing 0.1 M sodium fluoride to inhibit the DNase activity) for about 60 min. The vessel structures were isolated from the retinal cells by gentle rinsing in distilled water. After the vascular specimens were mounted on a slide, periodic acid-Schiff staining was performed. The specimens were then analyzed by microscope with digital capture (BX41 microscope, Olympus, Tokyo, Japan). The number of acellular capillaries per mm^2^ of capillary area was determined by counting 10 randomly selected microscopic fields.

### Immunofluorescence Staining

The trypsin digests were immunofluorescently stained as previously described [18]. The slides were incubated with a mouse anti-NG2 antibody (Chemicon, Temecula, CA, USA) for 1 h. To detect NG2, the slides were incubated with a fluorescein isothiocyanate (FITC)-conjugated goat anti-mouse antibody (Santa Cruz). The antibodies against NG2 label the somas of pericytes [47]. The number of pericytes present in 5 randomly selected fields was determined by counting the number of NG-2 positive cells per mm^2^ of capillary area.

### Fluorescein-dextran Microscopy

The fluorescein-dextran perfused retina was flat-mounted on a slide without fixation. The intact flat-mounted retina was then examined by fluorescence microscopy (Olympus). The status of vessel abnormality was determined using the following three parameters: the presence of an avascular area, fluorescence leakage and vessel narrowing. The parameter was scored from 0 (none) to 4 (severe).

### Quantification of AGEs Formation

To determine AGEs formation, serum and vitreous samples were analyzed in a competitive enzyme-linked immunosorbent assay (ELISA). The assay was performed using a monoclonal AGEs antibody (6D12, Cosmo bio, Tokyo, Japan) according to established protocol [48]. To evaluate the accumulation of AGEs in the retina, immunohistochemical staining was performed as previously described [37]. Antibodies were mouse anti-AGEs (Cosmo bio). For detection of AGEs, the sections incubated with LSAB kit (DAKO, CA, USA) and visualized by 3,3′-diaminobenzidine tetrahydrochloride.

### Apoptosis Assay

To evaluate apoptosis in retinal vascular cells, the TUNEL assay was performed with a kit (DeadEnd apoptosis detection system, Promega, Madison, WI, USA) according to the manufacturer’s instructions. Apoptotic cells were detected with peroxidase conjugated streptavidin in the retinal vascular digests. For quantitative analysis, TUNEL-positive nuclei were then counted in equal area of each slide.

### Southwestern Histochemistry for Detection of Activated NF-κB

To localize the NF-κB activity in the retinal vessels, in situ southwestern histochemistry was performed as described by Hernandez-Presa et al. [49]. As negative controls, the following were used: 1) absence of probe, 2) mutant NF-κB probe labeled with digoxigenin and 3) competition assays with a 200-fold excess of unlabeled NF-κB followed by incubation with labeled probe.

### Evaluation of NF-κB DNA Binding Using the ELISA-based Method

Nuclear proteins were isolated from retinas by using commercially available nuclear and cytoplasmic extraction kits (NE-PER™ Nuclear and Cytoplasmic Extraction Reagents, Pierce, IL, USA). NF-κB DNA-binding activity was evaluated using an ELISA-based EZ-detected™ Transcription Factor Kit for NF-κB p65 (Pierce) according to the manufacturer’s instructions. The amount of active NF-κB p65 in each sample was obtained from a standard curve and normalized to the protein content.

### Western Blotting Analysis in Retinal Tissues

Proteins were extracted from retinal tissues, and then 20 µg of protein lysates was separated by SDS–polyacrylamide gel electrophoresis and transferred to nitrocellulose membranes (Biorad, CA, USA). Membrane was probed with mouse anti-iNOS antibody (Santa Cruz), mouse anti-Bax antibody (Santa Cruz) and mouse anti-cIAP antibody (Santa Cruz), and then the immune complexes were visualized with an enhanced chemiluminescence detection system (Amersham Bioscience, NJ, USA).

### Statistical Analysis

Statistical evaluation of the results was performed using Student’s *t*-test and a one-way analysis of variance (ANOVA) followed by Tukey’s multiple comparison test using GraphPad Prism 4.0 software (Graph pad, CA, USA).

## Supporting Information

Figure S1 **Annexin V and propidium iodide staining of AGE-BSA-treated retinal pericytes.** Live retinal pericytes after treatment AGE-BSA with or without KIOM-79 were stained with Annexin V and propidium iodide (PI) and analyzed by flow cytometry as described under Materials and Methods. Early apoptotic cells were defined as Annexin V-FITC+/PI−, while necrotic cells were double-positive. 30,000 cells were analyzed in each case. A representative experiment is shown.
(TIF)

Figure S2 **Diabetes-induced occludin loss.** Representative retinal vessels from a normal Zucker lean rat (ZL), vehicle-treated ZDF rat (ZDF) and ZDF rat treated with KIOM-79 (KIOM-79) were stained with anti–occludin antibody. Occludin expression (red) was evident at the interfaces between adjacent endothelial cells in the control retinas, while it was mostly eliminated from the microvessels in the vehicle-treated ZDF rats.
(TIF)
