# Supplementary figures and images for: KIOM-79 Protects AGE-Induced Retinal Pericyte Apoptosis via Inhibition of NF-kappaB Activation In Vitro and In Vivo
Source: PLoS One. 2012 Aug 20;7(8):e43591. doi: 10.1371/journal.pone.0043591 (PMC3423361; doi:10.1371/journal.pone.0043591)

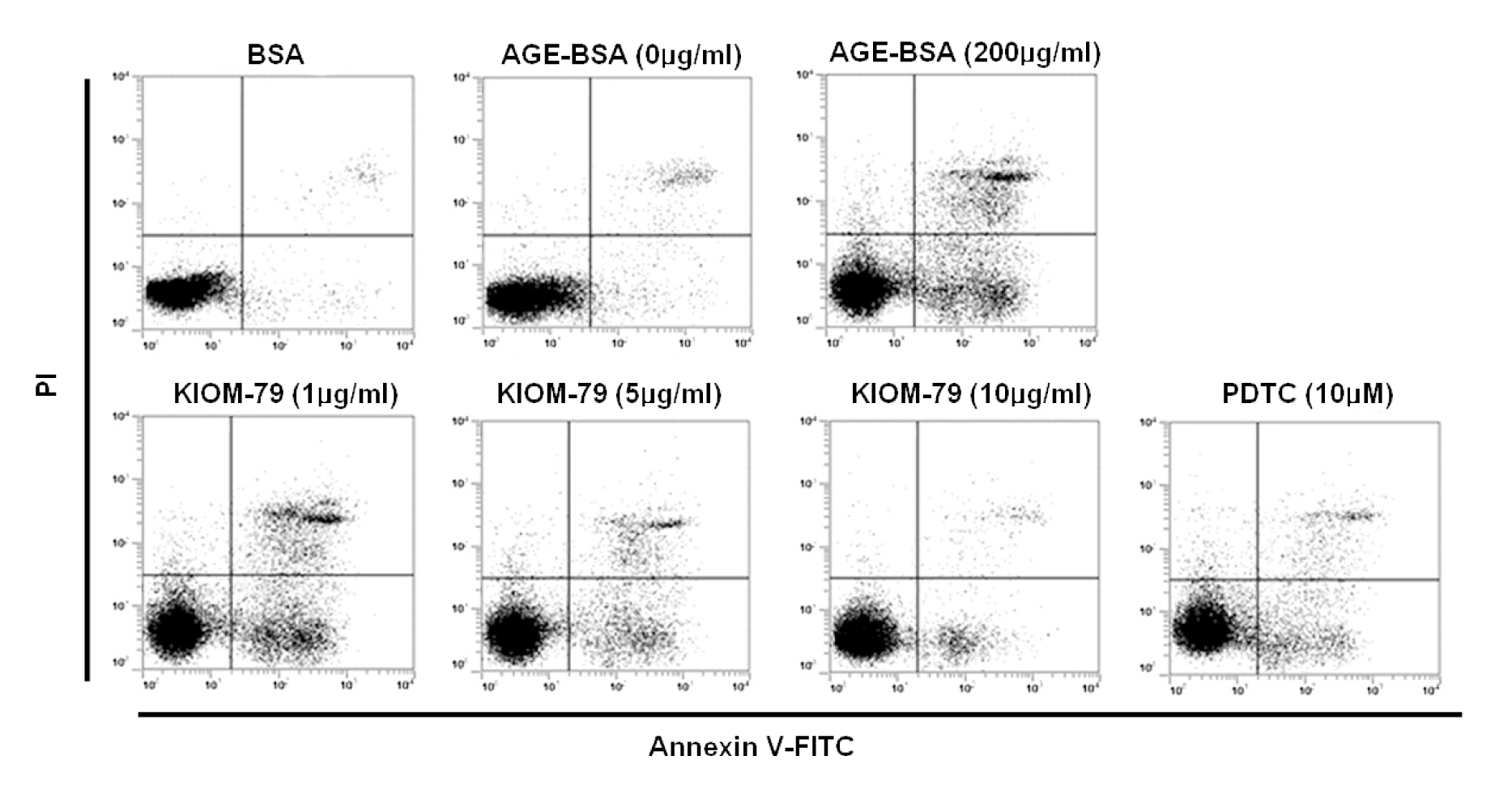

Supplement: Figure S1 — Annexin V and propidium iodide staining of AGE-BSA-treated retinal pericytes. Live retinal pericytes after treatment AGE-BSA with or without KIOM-79 were stained with Annexin V and propidium iodide (PI) and analyzed by flow cytometry as described under Materials and Methods. Early apoptotic cells were defined as Annexin V-FITC+/PI−, while necrotic cells were double-positive. 30,000 cells were analyzed in each case. A representative experiment is shown. (TIF) [file pone.0043591.s001.tif]

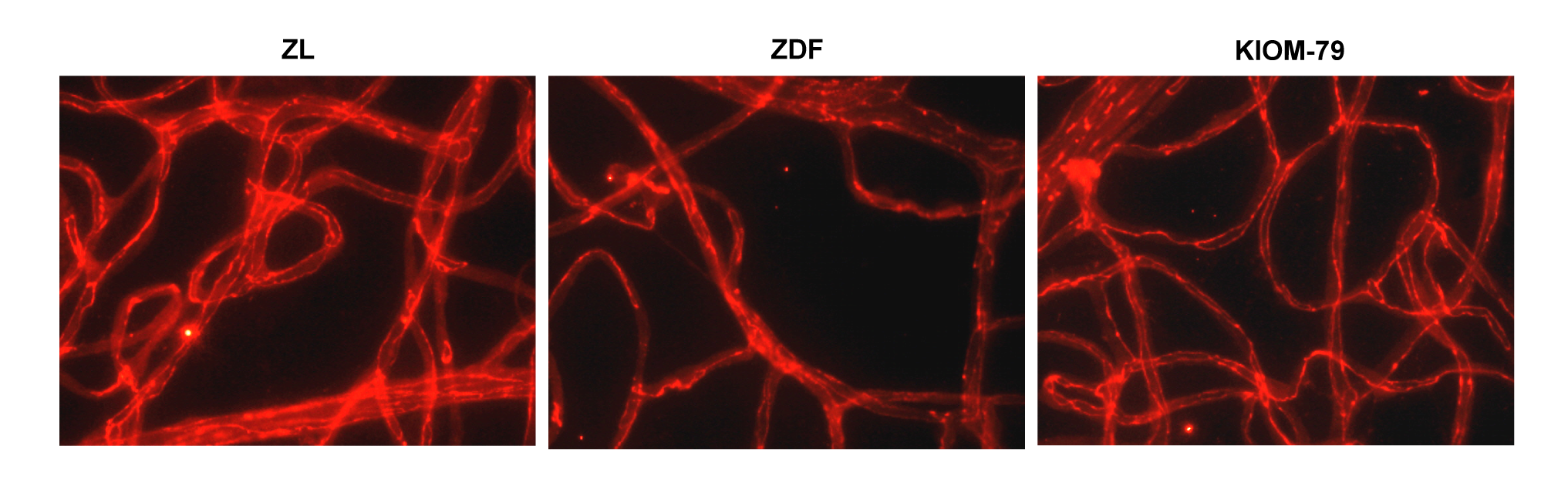

Supplement: Figure S2 — Diabetes-induced occludin loss. Representative retinal vessels from a normal Zucker lean rat (ZL), vehicle-treated ZDF rat (ZDF) and ZDF rat treated with KIOM-79 (KIOM-79) were stained with anti–occludin antibody. Occludin expression (red) was evident at the interfaces between adjacent endothelial cells in the control retinas, while it was mostly eliminated from the microvessels in the vehicle-treated ZDF rats. (TIF) [file pone.0043591.s002.tif]
